# Principles and Applications of Dual-Layer Spectral CT in Gastrointestinal Imaging

**DOI:** 10.3390/diagnostics13101740

**Published:** 2023-05-15

**Authors:** Paolo Niccolò Franco, Chiara Maria Spasiano, Cesare Maino, Elena De Ponti, Maria Ragusi, Teresa Giandola, Simone Terrani, Marta Peroni, Rocco Corso, Davide Ippolito

**Affiliations:** 1 Department of Diagnostic Radiology, Fondazione IRCCS San Gerardo dei Tintori, Via Pergolesi 33, 20900 Monza, Italy; francopaoloniccolo@gmail.com (P.N.F.); maria.ragusi@gmail.com (M.R.); teresagiandola1990@gmail.com (T.G.); r.corso@asst-monza.it (R.C.); davide.atena@tiscali.it (D.I.); 2 Department of Diagnostic Radiology, Istituti Clinici Zucchi, Via Zucchi 24, 20900 Monza, Italy; chiaraspasiano@gmail.com; 3 Department of Medical Physics, Fondazione IRCCS San Gerardo dei Tintori, Via Pergolesi 33, 20900 Monza, Italy; elena.deponti@unimib.it; 4 Philips Healtcare, Viale Sarca 54, 20126 Milano, Italy; simone.terrani@philips.it (S.T.); marta.peroni@philips.it (M.P.); 5 School of Medicine, Università Milano-Bicocca, Piazza dell’Ateneo Nuovo, 1, 20100 Milano, Italy

**Keywords:** image interpretation, computer-assisted, tomography, X-ray-computed, diagnostic techniques, digestive system

## Abstract

The advance in technology allows for the development of different CT scanners in the field of dual-energy computed tomography (DECT). In particular, a recently developed detector-based technology can collect data from different energy levels, thanks to its layers. The use of this system is suited for material decomposition with perfect spatial and temporal registration. Thanks to post-processing techniques, these scanners can generate conventional, material decomposition (including virtual non-contrast (VNC), iodine maps, Z-effective imaging, and uric acid pair images) and virtual monoenergetic images (VMIs). In recent years, different studies have been published regarding the use of DECT in clinical practice. On these bases, considering that different papers have been published using the DECT technology, a review regarding its clinical application can be useful. We focused on the usefulness of DECT technology in gastrointestinal imaging, where DECT plays an important role.

## 1. Introduction

Conventional single-energy computed tomography (SECT) is a diagnostic imaging technique that uses a polyenergetic X-ray beam from a single source that rotates around the patient’s body and a panel of detectors that records the radiation attenuated by the different densities of tissues, expressed in terms of Hounsfield Unit (HU).

Due to its fast acquisition and diagnostic accuracy, SECT has become the gold standard for the detection and assessment of different pathological entities. One of the limits of conventional SECT is that the characterization of tissues with a similar density is not always straightforward as, for instance, in the case of calcified plaques and iodinated blood within arterial vessels in angiographic studies. Moreover, SECT protocols frequently consist of repeated scanning before, during and after contrast injection, resulting in high-dose exposures.

Dual-energy CT (DECT) is a more recent technology that helps to overcome these limitations by acquiring data at two different energy levels to derive different tissue attenuations. Data obtained can be combined to generate images for routine clinical interpretation or more accurate material characterization [1].

The main contributors to attenuation coefficients during CT scanning are the photoelectric effect and the Compton scattering. Whereas the latter is minimally dependent on photon energy and is mainly related to a material’s electron density, the photoelectric effect is strongly X-ray-energy-dependent and increases with a higher element’s atomic number (Z). The photoelectric effect can be calculated by comparing attenuation levels derived from two energy levels. Because of its dependency on Z, it is crucial for distinguishing different materials with similar attenuation in any energy level. This characteristic is defined as material decomposition and represents the basis for spectral proprieties in DECT imaging [2]. Elements with high Z, such as iodine (Z = 53) or calcium (Z = 20), are susceptible to the photoelectric effect and have strong spectral properties. These elements present similar CT attenuation values in SECT due to their relative density.

Conversely, when exposed to different energy levels via DECT scanning, they interact in different ways, regardless of their density. This capability of differentiating structures with similar densities but different elemental compositions underlie multiple clinical applications of DECT scanning [3]. On the contrary, soft-tissue anatomic structures, including muscles or parenchyma, have a low photoelectric effect and consequently demonstrate less variability in their attenuation values at different energy levels.

The datasets of the two energy levels can be obtained using multiple acquisition techniques [4]. Depending upon how the two different X-ray energies are generated, DECTs are divided into two major groups: tube-based and detector-based. Two of the three leading DECT platforms currently in the market are tube-based: dual-source DECT (ds-DECT) (Somatom Drive/Somatom Definition Flash, Siemens Medical Solutions, Forchheim, Germany) and rapid kV-switching DECT (rs-DECT) (Revolution CT, GE Healthcare, Milwaukee, WI, USA; Aquilion ONE GENESIS Edition, Canon Medical Systems, Otawara, Japan). In the detector-based category, the dual-layer detector DECT (dl-DECT) (IQon spectral CT, Philips Healthcare, Eindhoven, The Netherlands) is the only currently available platform.

The first DECT scanner approved for clinical use was introduced into the market in 2006 and was based on a dual-source technique. These scanners consist of two detectors and two X-ray sources, a low-kV and a high-kV tube, with 90° orientation differences that scan simultaneously to achieve two energy spectra. Conversely, rs-DECT uses a single X-ray tube that rapidly alternates between low and high kV during its rotation (fast switching) and a single detector that registers information from both energies. The most recent technology is the ds-DECT, which was commercially introduced in 2016. It is based on a single energetic radiation tube associated with a detector panel, constituting two layers (sandwich detector) that simultaneously detect two energy levels.

This review aims to summarize the technical features of CT scanners with dual-layer detector technology, showing the added diagnostic value in daily practice of this approach via a review of the most recent literature on gastrointestinal applications.

### 1.1. Dual-Layer Detector Dual-Energy CT Technology

**Figure 1:**
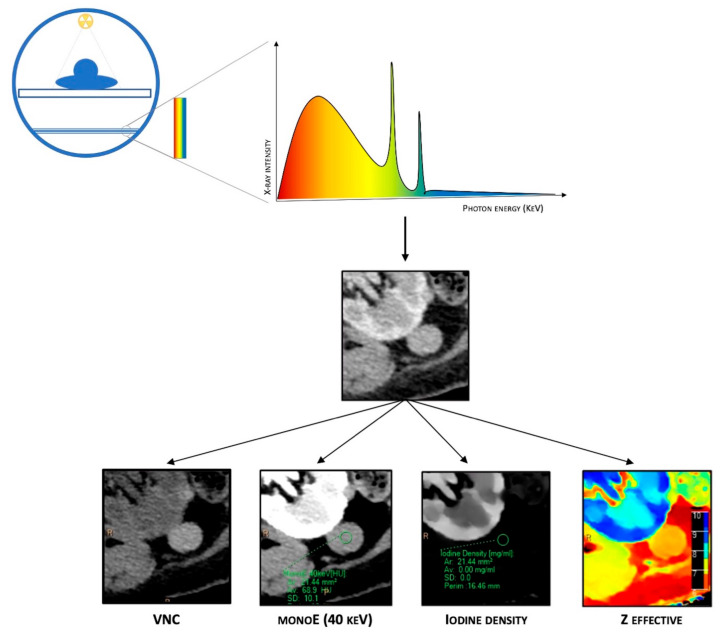
Schematic representation of DECT. It is based on a single energetic radiation tube associated with a detector panel constituted of two layers (sandwich detector) that simultaneously detect two energy levels. Different post-processing techniques are available due to spectral properties, such as material composition images (virtual non-contrast (VNC)), iodine maps, Z-effective imaging, and virtual monoenergetic images (VMIs).

As mentioned before, in the dl-DECT scanner system, spectral separation is achieved at the detector level. This system takes advantage of the polychromatic nature of the beam produced with a single-energy source, combined with highly specialized detectors that consist of two layers with maximal sensitivity for different energies. The top (inner) layer preferentially absorbs low-energy photons by design, approximately 50% of the total incident photon flux. In contrast, the bottom (outer) layer absorbs the remaining photons, which are primarily high-energy ones [5,6] (Figure 1).

A significant advantage of this system is, firstly, its excellent temporal registration. This system is well suited for material decomposition in the projection domain, making it quantitatively accurate and robust for possible patient motion. Another advantage is the perfect spatial registration of the acquired data to create a complete spectral dataset. The tube always operates at a high kVp, resulting in a high total X-ray power, which is advantageous for larger patients. Moreover, with this approach, scanning is performed at the full field of view of 50 cm. The last advantage is the dl-DECT retrospective acquisition mode: a dl-DECT scanner always acquires scans in the DECT mode, allowing one to gain spectral information for all scans performed, and hence there is no need to prospectively decide which scans perform in spectral mode, which is mandatory in other currently available dual-energy technologies. Retrospective on-demand spectral data of a region of interest allow radiologists to further investigate incidental findings without additional radiation exposure [5,7].

The main disadvantage of this system is its lower energy separation because the scintillator absorption properties do not offer a sharp distinction between lower- and higher-energy photons. As a result, the material differentiation contrast is decreased unless a higher radiation dose is used.

### 1.2. Dual-Layer CT Post-Processing

Combining data from both layers of detectors, dl-DECT scanners can generate conventional images comparable to those obtained from SECT, providing morphological details and material-specific image sets. Furthermore, plenty of different post-processing techniques are available due to spectral properties, such as material composition images (virtual non-contrast (VNC), iodine maps, Z-effective imaging, and uric acid pair images) and virtual monoenergetic images (VMIs). VNC images, also called “water-based”, are similar to conventional unenhanced CT images but are obtained via a dedicated algorithm that subtracts iodine-containing pixels from enhanced phases, allowing to create virtual plane images. Iodine concentration (IC) images (iodine maps) are material decomposition maps obtained via an algorithm that enhances only the pixels containing iodine. Iodine maps allow for identifying the presence or absence of iodine and its uptake in several tissues, which is particularly helpful in evaluating the contrast enhancement. Z-effective imaging consists of colorimetric maps that visually enhance the differences between tissues: the average atomic numbers of elements in each pixel are translated into color-coded images that provide a higher degree of discrimination than HU attenuation in conventional CT. Z-effective mapping is also used to define the peak enhancement (PE), which expresses the maximal concentration of the contrast agent with time in a tissue, according to the acquisition phase. Uric acid pair images show only pixels containing uric acid with original HU values, while all others appear dark, which is extremely useful for assessing urinary calculi composition and gout.

Finally, VMIs are a set of monochromatic images that simulate the appearance of images acquired using a monoenergetic X-ray beam at a selected energy level. VMIs can be obtained at discrete energy levels ranging from 40 to 190 keV with dl-DECT. Due to the approximation of the energy with the K-edge of iodine, low-keV VMIs show increased iodine conspicuity, which results in attenuation values equivalent to conventional images at a 120-kVp, but with a significant reduction in noise. Conversely, higher energy levels in VMIs reveal decreased iodine conspicuity and a drop in beam hardening artifacts, a physical phenomenon of the beam itself that produces an artifact that typically appears in the presence of metallic implants.

### 1.3. Radiation Dose

The White Paper of the Society of Computed Body Tomography on Dual-Energy CT published in 2016 stated that DECT acquisitions, even if using different X-ray spectra, do not provide additional radiation dose exposure in patients [8].

In the literature, various studies have demonstrated similar or lower radiation dose exposure via DECT acquisitions compared to SECT [9,10,11,12]. One investigation revealed that DECT imaging at 80 and 140 kVp resulted in a decrease in the dose-length product and CT dose index values of 10% and 12%, respectively, compared to standard SECT (120 kVp) imaging using the same dual-source scanner, with no significant difference in objective image noise or subjective image quality [9]. Duan et al. compared radiation dose and image quality for abdominal CT imaging performed on dl-DECT and conventional SECT scanners in patients of different sizes. The volume CT dose index (CTDIvol) during dl-DECT was similar to one measured on a conventional SECT for average-size patients, lower for smaller patients, and slightly higher for larger patients [11].

Furthermore, VNC imaging allows one to reconstruct plain images from enhanced phases, reducing the number of scans and, consequently, the radiation dose [13]. This tool is particularly advantageous in oncologic patients, who usually undergo repeated follow-up CT examinations, and pediatric patients.

Finally, a potential radiation dose reduction can be achieved by avoiding additional CT studies for further incidental lesion characterization.

## 2. Clinical Applications

Table 1 gathers published papers regarding current evidence on the utility of dl-DECT for gastrointestinal imaging.

### 2.1. Liver

The added value of DECT technology in hepatic imaging mainly consists of helping radiologists visualize and correctly characterize lesions and quantify the degree of diffuse hepatic diseases.

Regarding focal liver lesions, it has been shown that DECT low-energy VMIs facilitate and improve the assessment of hypervascular lesions [14,15]. Preliminary research on dl-DECT platforms has demonstrated similar results. Große Hokamp et al. observed that, throughout the entire keV spectrum, VMIs at 40 keV had the highest detectability of arterially hyper-enhancing lesions in phantoms and in vivo due to an increase in lesion contrast without an increase in image noise [16]. Furthermore, in a more recent investigation, low-energy VMIs also improved the wash-out assessment of arterially hyper-enhancing liver lesions in contrast-enhanced dl-DECT scans. The authors evaluated a population of patients undergoing CT scans for hepatocellular carcinoma (HCC) screening. Both wash-out assessment and image quality parameters resulted in significantly better VMIs at 40 kV compared to higher-energy VMIs and conventional CT imaging [17]. The significant advantage of low-energy VMIs was also proven concerning the assessment of hypovascular liver metastases. Nagayama et al. demonstrated that both the tumor-to-liver contrast and contrast-to-noise ratio (CNR) increased as the energy decreased. At the same time, 40 kV VMIs overcame higher-energy VMIs and PEI in lesion detectability [18].

DECT can also be considered as a helpful approach for diffuse liver diseases. An accurate evaluation of liver fibrosis is clinically significant due to its correlation with carcinogenesis and prognosis. The degree of fibrosis is conventionally assessed via blood tests, ultrasonography-based transient elastography, and magnetic resonance elastography [19]. Many studies have investigated the applications of tube-based DECTs in the assessment of liver fibrosis, mainly via the measurement of parenchymal IC on equilibrium imaging and the quantification of liver extracellular volume (ECV) [20,21,22]. In 2021, Morita et al. evaluated these measurements for the first time using a dl-DECT platform. The authors demonstrated that the iodine density ratio (calculated by dividing the iodine density of the liver parenchyma by the iodine density of the aorta) and the CT-ECV increased significantly as the fibrosis stage advanced (*p* < 0.01 for both). The CT-ECV showed better diagnostic accuracy for the degree of fibrosis. In the case of advanced-stage fibrosis, the sensitivity ranged from 90% to 95%, and the specificity ranged from 72.9% to 85.4% among two readers [23].

Dl-DECT may also improve the assessment of iron overload. Ma et al. compared the evaluation of liver and cardiac iron overload with T2*-weighted unenhanced MRI to unenhanced dl-DECT scans in patients with myelodysplastic syndromes and aplastic anemia. The two techniques were comparable in the case of iron overload in the liver [24].

The capability of DECT to derive VNC images from contrast-enhanced examinations by identifying and subtracting iodine potentially underlies many applications in liver imaging, particularly concerning liver steatosis. Different studies have compared the performance in diagnosing fatty liver between true non-contrast (TNC) images and VNC images generated from DECT scanners [25,26]. In their study, Choi et al. proved that, even if liver densities in VNC images were significantly different from those in TNC images, various parameters (liver and spleen densities, liver-minus-spleen density, and liver-to-spleen ratio) would be significantly higher in healthy liver patients than in fatty liver patients in TNC images as well as in VNC images from multiple phases. Additionally, the diagnostic performances of all parameters for fatty liver diagnosis in VNC images were not significantly different to those in TNC images [25].

To our knowledge, there is a lack of publications regarding the application of dl-DECT to assess liver steatosis. However, the value of VNC imaging obtained using a dl-DECT platform for the assessment of liver attenuation has been compared to conventional TNC images. Laukamp and colleagues demonstrated that VNC images from various enhanced phases were not significantly different from the TNC ones in terms of liver attenuation and image noise. However, the accuracy decreased in the early arterial phases of the liver when only a small quantity of contrast media was present in the parenchyma [27,28].

Finally, in recently published research, the authors described another technical advantage of dl-DECT platforms. They found that peristalsis-related artifacts were significantly less frequent and less relevant when the liver was evaluated with iodine image reconstructions than with conventional 120 kVp images in both qualitative and quantitative analysis [29].

### 2.2. Gallbladder and Biliary Tree

**Figure 2:**
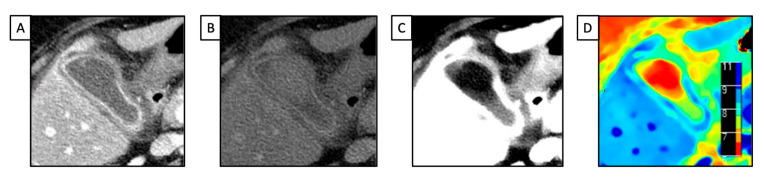
A 42 y-o patient with right upper pain underwent abdominal CT with a final diagnosis of cholecystitis. (**A**) Conventional CT image acquired after intravenous contrast media injection shows diffuse thickening of the gallbladder wall, without evidence of any calcific stone; (**B**) low mono-energetic map shows a hypoattenuating round stone, due to cholesteric composition; (**C**) opposite that, the high mono-energetic map demonstrates the hyperattenuating mass consistent with the cholesteric gallstone; (**D**) the Z-effective map allows us to better define the different structures of the images via the different atomic values of the gallbladder: blue (contrast agent), red (lipid content), and green (fluid).

In CT diagnosis, gallbladder stones are indicated directly via high-density or low-density stones and indirectly via the dilation of the intrahepatic biliary duct, left and right hepatic ducts, biliary duct, and gallbladder [30]. Based on density, gallbladder stones are classified into high-, iso-, low-, and mixed-density stones, relative to the density of the surrounding bile. Conventional SECT has high accuracy in diagnosing high-density or low-density gallbladder stones [31]. However, it is challenging to diagnose iso-density stones, such as those made of cholesterol, due to their similar attenuation value with the bile (Figure 2).

DECT provides a new approach for the differential diagnosis of gallbladder stones and reliable information for their clinical treatment. In 2018, Saito et al. applied the property of dl-DECT to provide on-demand retrospective spectral analysis to detect iso-dense biliary stones that were not detected using conventional CT scans. Using magnetic resonance cholangiopancreatography (MRCP) or endoscopic retrograde cholangiopancreatography (ERCP) as the reference standard, the authors found that in two out of three cases, the stones were readily detected in VMIs at 40 keV. In contrast, only a small stone (<5 mm) remained undetected during spectral dataset evaluation [32].

In their prospective ex vivo study, Soesbe and colleagues devised a method for detecting iso-dense stones using a dl-DECT scanner. They compared it with previously reported methods built using tube-based DECT images. After placing iso-dense gallstones inside vials containing ox bile, six readers evaluated the presence of isodose gallstones via conventional PEI at 120 kVp, VNC images, VMIs at 40 and 200 keV 120 kVp, and segmented images obtained using a two-dimensional histogram of Compton and photoelectric X-ray attenuation derived from dl-DECT. The authors found that for gallstones measuring less than 9 mm, the segmented images had the highest overall AUC (*p* < 0.01) compared to VMIs [33].

Only one study published in the literature evaluated the potential role of dl-DECT technology in evaluating acute cholecystitis. In this research, spectral imaging obtained using a dl-DECT and an rs-DECT were compared with SECT imaging for the detection of multiple individual findings associated with acute cholecystitis, such as gallbladder fossa hyperemia, gangrene, and heterogeneous wall enhancement. During the evaluation of two readers, DECT showed increased sensitivity (R1, 86%; R2, 89.5%) compared with conventional CT (R1, 77.2%; R2, 70.2%) for the diagnosis of acute cholecystitis and the assessment of the aforementioned related findings [34].

### 2.3. Pancreas

The CT appearance of most focal pancreatic lesions usually ranges from mildly hypo- to iso-attenuating in comparison with the normal pancreatic parenchyma, making the differential diagnosis a radiological dilemma [35]. Several investigations demonstrated that spectral imaging could improve the diagnosis of pancreatic tumors in terms of lesion conspicuity, extension, and vascular invasion [36,37].

Even despite its more recent commercialization, some studies have already focused on the application of dl-DECT technology for diagnosing pancreatic lesions. In a cohort of 61 patients with different types of pancreatic lesions, El Kayal et al. found that, compared to conventional poly-energetic imaging (PEI), low-energy VMIs and iodine maps facilitated subjective lesion delineation, because of the increased attenuation of iodine at energy levels close to its maximum absorption (33 keV). Moreover, dl-DECT imaging increased reader diagnostic confidence, assessed using a five-point Likert scale by two radiologists [38]. In patients with pancreatic ductal adenocarcinoma (PDAC), detector-based MVI at 40 KeV yielded better quality lesion assessment in each enhancement phase than PEI due to its high pancreas tumor contrast and vascular opacification without a relevant increase in image noise [39]. In a more recent publication, Han and colleagues compared portal-venous-phase VMIs obtained using a dl-DECT scanner with the traditional polychromatic pancreatic phase scan for the diagnosis of PDAC. The authors found that low-energy VMIs at 40 and 55 KeV had a higher tumor-to-pancreas contrast-to-noise ratio (CNR), attenuation difference, and higher peripancreatic vascular CNR and signal-to-noise ratio (SNR) than the pancreatic phase image (*p* < 0.001). Furthermore, in a subjective analysis, VMIs at 55 KeV showed the best tumor conspicuity [40].

Not so many studies have been performed to evaluate neuroendocrine neoplasms (NENs) of the pancreas. In their research, Wang et al. analyzed both DECT (NIC, tumor attenuation, and effective Z) and SECT features in a cohort of 104 patients with pathologically confirmed NEN. The authors found that combining DECT metrics (NIC, tumor attenuation, and effective Z) with qualitative SECT features improved the differential diagnosis between neuroendocrine tumors and neuroendocrine carcinomas [41].

### 2.4. Gastrointestinal Tract

**Figure 3:**
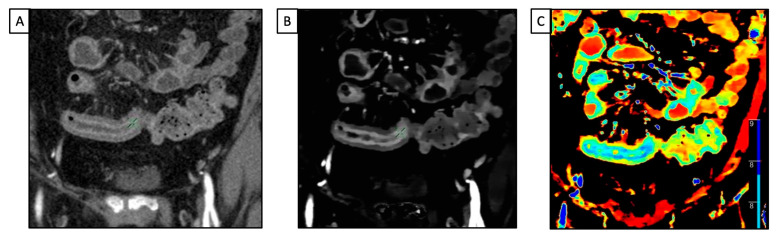
A 49 y-o male with known Crohn’s disease, diffuse abdominal pain, and suspected relapse of disease underwent abdominal CT. (**A**) Conventional CT images on the coronal plane acquired after intravenous contrast media injection show a poor layered enhancement appearance of the distal ileum; (**B**) iodine map enhances iodine’s uptake by the mucosa layer, and the hypoattenuating appearance of the submucosa one, consistent with edema; (**C**) the Z-effective map allows us to define the pattern of enhancement due to the atomic number of iodine.

DECT has proven to have many applications, including in the evaluation of gastrointestinal tract diseases. In emergency settings, CECT is typically the preferred imaging tool when acute bowel ischemia is suspected to assess a decreased or lack of bowel parietal enhancement. However, detecting these findings is not always straightforward, especially in the case of early ischemia [42]. DECT can improve confidence in diagnosing bowel ischemia due to its capability in enabling a quantitative measure of wall enhancement via iodine mapping. In addition, a low-keV VMI can highlight the attenuation differences between perfused and non-perfused walls [43,44] (Figure 3).

Among the different spectral technologies, dl-DECT is particularly useful because it allows for the simultaneous acquisition of low- and high-KeV information at the same spatial position, facilitating the visualization of the un-enhanced bowel, and because the spectral dataset is always retrospectively available for every scan [45].

Possible applications of DECT, iodine mapping, and VMIs for the evaluation of gastrointestinal cancers have been explored in various pieces of research [46]. In Chen and colleagues’ investigation, quantitative parameters extracted via dl-DECT imaging (iodine concentration (NIC), slope of the spectral HU curve, and effective Z) showed a significant correlation with both pT stages and two histologic-grade groups of colorectal adenocarcinomas [47]. Wang et al. verified that the IC and NIC generated from dl-DECT imaging could help to detect local colonic wall thickening caused by colon neoplasia among radiologically indeterminate colonic wall thickenings, with no specific requirement for bowel distension or luminal insufflation [48].

**Figure 4:**
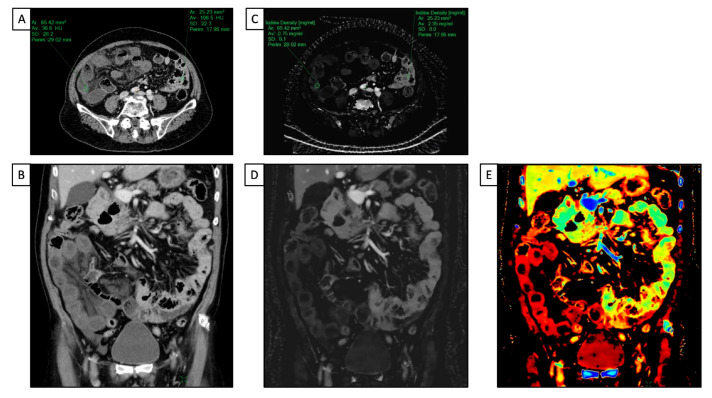
A 66 y-o male with diffuse abdominal pain underwent abdominal CT with a final diagnosis of distal ileum bowel ischemia. (**A**,**C**) Axial and coronal conventional CT images acquired after intravenous contrast media injection show a slight difference in attenuation value (HU) of the small bowel walls, with reduced enhancement in the distal ileum and regular enhancement in jejunum and proximal ileum; (**B**,**D**) iodine maps clearly show the difference in iodine uptake between the normal walls of the jejunum and proximal ileum and the poor iodine uptake of the distal tract of the ileum; (**E**) the Z-effective map allows us to better and more simply define the different enhancement in terms of colors between healthy and ischemic bowels due to the atomic number of iodine.

Even if MRI is considered the gold standard in evaluating small intestine inflammatory bowel diseases [Maaser2019], DECT has proven to have high diagnostic accuracy in assessing inflammation activity and severity in those with Crohn’s disease [49,50,51] (Figure 4).

In particular, it has been demonstrated that a quantitative assessment of DECT parameters, including NIC and slope of the HU curve (which represents the X-ray attenuation coefficient at different energy levels), had higher accuracy in predicting intestinal activity and severity in ileocolonic Crohn’s disease when compared to conventional SECT parameters [49]. Further investigations corroborated these results and proved that the walls of the bowel segments with active inflammation show a higher NIC value than those without inflammation, using histopathologic results from either ileocolonic resection or biopsy of the terminal ileum as the reference standard [51].

With regard to detector-based platforms, Kim et al. evaluated various qualitative and quantitative dl-DECT features in a population of thirty-nine patients with Crohn’s disease at different stages, ranging from remission to severe activity state. Due to its ability to quantify the contrast distribution across intestine walls at a single point in time, the iodine concentration measured on the iodine map was the only independent variable associated with the Crohn’s disease activity index [52]. Active disease qualitative assessment may also be improved because of the increased hyper-enhancement seen in low-keV images. Lee et al. demonstrated that low-energy (40 keV) VMIs on a dl-DECT scan provided the best CNR for both healthy and pathologic small bowel walls. Moreover, the diagnostic performance in assessing active Crohn’s disease of three radiologists with different levels of experience was significantly improved with the addition of low-energy VMIs, compared to only the conventional PEI at 120 kVp [53].

CT colonography is a low-dose and minimally invasive method for diagnosing clinically relevant lesions within the colon lumen. However, electronic fecal cleansing is often needed to correct inadequate bowel preparation using fecal tagging with iodine or barium. In the literature, a possible advantage of the tube-based DECT approach for electronic cleansing in CT colonography imaging has been proposed [54]. Nevertheless, tube-based technology may lead to an increase in radiation dose and image noise at a lower keV. Conversely, dl-DECT allows for VMIs in the projection domain without the need for temporal and angular interpolation because it measures low- and high-energy projection information in the two layers of the detector at the same spatial and angular location. This may theoretically yield more accurate beam hardening artifact correction. In addition, since spectral data are always available, using dl-DECT fecal tagging density can be calibrated to a precise level after scanning. Taguchi et al. investigated these aspects, demonstrating that both the mean tagging density and the number of colon segments with appropriate tagging density were significantly higher in dl-DECT VMIs than that in conventional 120 kVp images (*p* < 0.01 for both) [55]. A more recent investigation further strengthened these promising results. The authors found that 40-keV monoenergetic images showed higher overall sensitivity in polyp detection compared with conventional 120 kVp PEI (58.8% vs. 42.1%, *p* < 0.001) and improved reader confidence at different fecal tagging levels (*p* < 0.001) in a colon phantom [56].

## 3. Conclusions

Dual-layer DECT has several applications in gastrointestinal imaging and has already demonstrated utility above that of conventional SECT in various pathologies. Particularly, virtual monoenergetic images can improve vascular contrast and lesion conspicuity at low energy levels. Iodine concentration measurements may help to characterize different lesions by highlighting their contrast enhancement. Virtual non-contrast imaging can provide pre-contrast information and potentially eliminate true non-contrast series in multiphasic studies, saving radiation doses and additional studies. Lastly, a significant advantage of this dual-layer DECT is that the spectral information is provided retrospectively for all patients, without the need for prospectively screening the patient to determine whether the dual-energy mode must be turned on.

## Figures and Tables

**Table 1:** Overview of the reviewed sources regarding dl-DECT applications in gastrointestinal imaging.

Author	Year	Country	Study Nature	Pathology	Number of Subjects
Liver					
GroßeHokamp	2018	Germany	Retrospective	Arterially hyper-enhancing liver lesions	20
Reimer	2021	Germany	Retrospective	Arterially hyper-enhancing liver lesions	31
Nagayama	2019	Japan	Retrospective	Hypovascular liver metastases	81
Morita	2021	Japan	Retrospective	Liver fibrosis	68
Ma	2020	China	Prospective	Liver iron overload	31
Gallbladder and biliary tree					
Saito	2018	Japan	Retrospective	Iso-dense biliary gallstones	3
Soesbe	2019	USA	Prospective	Iso-dense biliary gallstones	105
Huda	2021	USA	Retrospective	Acute cholecystitis	57
Pancreas					
El Kayal	2019	Germany	Retrospective	Pancreatic lesions (PDAC, cyst lesions, IPMN, MCN, NET, lymphomas, metastasis, chronic pancreatitis)	61
Nagayama	2019	Japan	Retrospective	PDAC	48
Wang	2022	China	Retrospective	Neuroendocrine neoplasms	104
Gastrointestinal tract					
Chen	2022	China	Retrospective	Colorectal cancer	131
Wang	2021	China	Retrospective	Colonic wall thickening	80
Lee	2018	Korea	Retrospective	Crohn’s disease	76
Kim	2018	Korea	Retrospective	Crohn’s disease	39
Taguchi	2018	Japan	Retrospective	Electronic cleansing	35

Dl-DECT: dual-layer detector dual-energy computed tomography; PDAC: pancreatic ductal adenocarcinoma; IPMN: intraductal papillary mucinous neoplasm; MCN: mucinous cystic neoplasm; NET: neuroendocrine tumor.

## Data Availability

Not applicable.
